# Efficacy of Dupilumab on Different Phenotypes of Atopic Dermatitis: One-Year Experience of 221 Patients

**DOI:** 10.3390/jcm9092684

**Published:** 2020-08-19

**Authors:** Simona Tavecchio, Luisa Angileri, Francesco Pozzo Giuffrida, Francesca Germiniasi, Angelo Valerio Marzano, Silvia Ferrucci

**Affiliations:** 1 Dermatology Unit, Fondazione IRCCS Ca’ Granda Ospedale Maggiore Policlinico, Via Pace 9, 20122 Milan, Italy; luisaangileri@hotmail.it (L.A.); francesca.grm@gmail.com (F.G.); angelo.marzano@unimi.it (A.V.M.); silviaferrucci@gmail.com (S.F.); 2 Department of Physiopathology and Transplantation, Università degli Studi di Milano, Via Pace 9, 20122 Milan, Italy; 3 Ophthalmological Unit, Department of Clinical Sciences and Community Health, University of Milan, Fondazione Cà Granda, IRCCS Ospedale Maggiore Policlinico, 20122 Milan, Italy; francescopozzo@yahoo.com

**Keywords:** atopic dermatitis, dupilumab, clinical phenotypes, quality of life, drug response

## Abstract

Background: The clinical features of adult-onset atopic dermatitis (AD) are heterogeneous and the diagnosis can be a challenge. A new biologic drug (dupilumab) has been approved for moderate to severe AD in adult patients. The efficacy and safety have been demonstrated in clinical trials, but these studies do not reflect conditions in daily practice and do not consider the different clinical manifestations of AD. Objectives: Analyzing the dupilumab activity in a real-world setting and comparing its efficacy on different AD phenotypes. Methods: We retrospectively evaluated 221 AD patients treated with dupilumab, stratified into six clinical phenotypes: classic, generalized eczema inflammatory and lichenoid patterns, prurigo, nummular eczema, and erythroderma. At baseline and at weeks 4, 16, and 52, the disease severity was assessed through the Eczema Area and Severity Index (EASI) and the quality of life was assessed through the Dermatology Life Quality Index (DLQI) questionnaire, Peak Pruritus Numerical Rating Scale (itch NRS), and Peak Sleep NRS. Results: We found a significant improvement after 16 weeks of treatment (*p* < 0.0001) in all six phenotypes for all the assessed scores mentioned above, persisting up to week 52. The best improvement was seen in the more severe phenotypes, particularly the erythrodermic one. Conclusions: The present study confirmed the efficacy and safety of dupilumab in the treatment of severe AD. Its strength was in the stratification of AD patients in six different phenotypes based on their clinical presentation, all of whom markedly improved in terms of both clinically evident and reported symptoms, as well as their quality of life.

## 1. Introduction

Atopic dermatitis (AD), or atopic eczema, is a chronic inflammatory skin disease affecting 7–10% of adults [1]. Onset is usually in early childhood, but it may persist into adulthood or present ex novo during this period [2,3,4,5]. Recently, the term adult-onset atopic dermatitis has been introduced with a lack of a clear acceptance in the literature; we can speculate that this is due to difficulties in diagnosing and classifying this disease. It is well known how to diagnose atopic dermatitis in children by using clinical criteria [6,7,8,9,10,11,12,13]. In cases of adult-onset AD, the clinical presentation, apart from the classical form, is either head-and-neck dermatitis, hand eczema, multiple areas of lichenification, or prurigo lesions [14,15]. Considering the lack of anamnestic dermatological manifestations, for this dermatitis, the diagnosis of AD can be a challenge. We think that it is important to focus on the different forms of AD in order to also benefit from the new treatments. A new biologic drug (dupilumab) was approved for moderate to severe AD in adult patients. It is a fully human monoclonal antibody directed against the alpha subunit of the interleukin-4 receptor, blocking the signaling of both type-2 cytokines IL-4 and IL-13. The efficacy and safety were demonstrated in clinical trials [16,17,18] but these studies do not reflect conditions in daily practice and do not consider different clinical presentations of AD. The aim of our study was to analyze the activity of dupilumab in a real-world setting by comparing the efficacy on different clinical phenotypes. Between November 2018 and February 2020, a total of 221 adult patients with severe AD (Eczema Area and Severity Index (EASI) ≥ 24), in which treatment with ciclosporine led to an inadequate clinical response or development of side effects or was controindicated, were enrolled from the Dermatology Unit of the Fondazione IRCCS Ca’ Granda Ospedale Maggiore Policlinico of Milan, Italy.

## 2. Materials and Methods

We evaluated 221 adult patients referred to our department from November 2018 to February 2020 that were affected by severe AD and treated with dupilumab. All patients were treated with 300 mg of self-administered subcutaneous dupilumab every other week following a loading dose of 600 mg of dupilumab subcutaneously administered by a clinician. Traditional immunosuppressive agents (e.g., cyclosporine, azathioprine, methotrexate) were discontinued at least 4 weeks before the dupilumab initiation in all patients, while systemic corticosteroids were maintained in a minority of patients, with progressive tapering and subsequent withdrawal within 2 weeks. Concomitant topical corticosteroids or calcineurin inhibitors were allowed. We divided all the patients into two groups: early-onset AD and adult-onset AD by considering whether the age of onset of AD was under or over 18 years. The diagnosis of adult-onset AD was made by considering the history of the patient and the clinical features using Hanifin and Rajka’s criteria [6], and in some cases, we performed other exams, such as patch testing, blood analysis, and/or a cutaneous biopsy to rule out other diseases. Moreover, we divided all the patients into six subgroups by considering the different clinical forms of AD according to the paper of Silvestre et al. [19]; we considered only six phenotypes of AD because in this study, we investigated only the patients treated with dupilumab, which is approved in Italy for only severe AD with an EASI ≥ 24; therefore, some of the phenotypes described by Silvestre et al. could not be considered because of their limited extension. Below, we report a description of their main features that we used to classify them: (i) lichenified/exudative flexural dermatitis, almost always associated with head-and-neck eczema and/or hand eczema, where in this form, a percentage of patients did not present the flexural pattern (about 10%) or had involvement of the extensor surface of the limbs or the trunk (about 30%); (ii) generalized eczema with an inflammatory pattern with diffuse erythema and was predominantly exudative, and crusted eczematous lesions; (iii) generalized eczema with a lichenoid pattern with lichenification, excoriations, crusts, and xerosis; (iv) prurigo with highly pruriginous papules and nodules; (v) nummular eczema with round, inflamed sores; and (vi) erythroderma with over 90% of the skin surface being red, dry, and lichenified. Clinical documentation was collected at baseline and after 4, 16, and 52 weeks from the beginning of the treatment using validated scores [20]: EASI and the Dermatology Life Quality Index (DLQI). Itch and sleep disorders were evaluated on a numerical rating scale (itch-NRS and sleep-NRS), as well as the peak value during the previous 72 h. The primary endpoint of our study was to analyze the efficacy of dupilumab in a real-world setting in terms of clinical improvement and quality of life impact by comparing the scores mentioned in the six phenotypes above in which the patients were stratified.

### Statistical Analyses

Data collected from electronic medical records included the mean ± standard deviation (SD), medians (Q1–Q3, first and third quartiles), and percentage. A chi-square test (Pearsons *χ*^2^ test) was used to assess the relationships between categorical variables in independent groups. Differences in the clinical index (EASI, DLQI, Pruritus NRS, and Sleep NRS) means between the six phenotype groups were compared using one-way ANOVA; LSD (least significant difference) post hoc tests were used because of the different numbers of patients in each group (Table 1). Changes in the index scores from baseline were analyzed in patients who completed a 16-week follow-up; we used a paired-samples *t*-test in order to determine whether the differences were significant. A *p*-value < 0.05 was considered statistically significant. All analyses were conducted using SPSS Statistics for Macintosh, version 20.0 software (IBM Corp., Armonk, NY, USA).

## 3. Results

### 3.1. Overview

We performed a retrospective study on 221 adult patients affected by severe AD and treated with dupilumab. This novel drug is approved for adult patients with severe AD in which treatment with cyclosporine led to an inadequate clinical response, the development of side effects, or was controindicated. In our cohort, 26 (11.76%) out of 221 patients had contraindications to cyclosporine, whereas 74 (33.48%) patients developed side effects during treatment with cyclosporine and 121 (54.75%) patients experienced an inadequate clinical response.

The distribution of the 221 AD patient’s clinical characteristics is reported in Table 2. Of these patients, 161 (72.85%) had early-onset AD and 60 (27.15%) had adult-onset AD.

In Table 3, we report the number of patients that reached 4 weeks of treatment (221), 16 weeks of treatment (154 patients), and 52 weeks of treatment (72 patients). The reduction of the number of patients that reached 16 and 52 weeks of treatment was due to the fact that patients started treatment at different times and they did not achieve 16 or 52 weeks of treatment at the moment of the publication of this paper. A very low number of patients dropped out of the treatment (6 out of 221 patients (2.71%)): one patient after 52 weeks because of inadequate response, one patient after 12 weeks of treatment due to the development of severe conjunctivitis that had a high impact on his job, one patient at 16 weeks because of the persistent elevation of their eosinophils count (>20,000/μL) despite the introduction of prednisone, one patient at 12 weeks because of a severe elevation of eosinophils (>5000/μL) and severe blepharitis and conjunctivitis, one patient after 40 weeks because of the absence of a response, and one patient after 16 weeks because of the development of severe psoriasis.

In Table 3, we give the mean EASI score, the mean DLQI score, their percentages of reduction, and EASI75 (a 75% reduction from baseline in the EASI score) at 4, 16, and 52 weeks of treatment of all 221 patients.

At baseline, the mean EASI score was 30.73 (SD: 7.93) and the mean DLQI was 16.41 (SD: 6.73).

After 4 weeks of treatment, the mean EASI score was 8.66, the mean DLQI score was 7.02, and EASI75 was reached by 49.77% of patients.

After 16 weeks of treatment, the mean EASI score was 5.29 (SD: 4.82; *p* < 0.0001), the mean DLQI score was 4.72 (SD: 4.75; *p* < 0.0001), and EASI75 was reached by 75.97% of patients.

Lastly, after 52 weeks of treatment, the mean EASI score was 4.59, the mean DLQI score was 2.8, and EASI75 was reached by 83.33% of the patients.

### 3.2. AD Phenotypes

#### 3.2.1. Classic: Lichenified/Exudative Flexural Dermatitis, Almost Always Associated with Head-and-Neck Eczema and/or Hand Eczema (101 Patients)

The mean EASI score was 27.86 at baseline and it decreased to 6.64 at week 4, to 4.27 at week 16, and to 3.1 at week 52 (reduction percentages were 76.17%, 84.67%, and 88.87%, respectively). A total of 68.89% of patients achieved EASI75 at week 4, 81.54% at week 16, while at week 52, the percentage of patients who achieved EASI75 raised to 95%.

The mean DLQI score was 16.58 at baseline and it decreased to 7.17 at week 4, to 4.48 at week 16, and to 2.71 at week 52 (reduction percentages were 56.76%, 72.98%, and 83.65%, respectively).

The mean itch-NRS score was 8.47 at baseline and it decreased to 3.94 at week 4, to 3.36 at week 16, and to 2.43 at week 52 (reduction percentages were 53.48%, 60.33%, and 71.31%, respectively).

The mean sleep-NRS score was 7.08 at baseline and it decreased to 2.04 at week 4, to 1.36 at week 16, and to 0.73 at week 52 (reduction percentages were 71.19%, 80.79%, and 89.69%, respectively).

#### 3.2.2. Generalized Eczema with an Inflammatory Pattern (41 Patients)

The mean EASI score was 31.78 at baseline and it decreased to 10.26 at week 4, to 6.64 at week 16, and to 4.5 at week 52 (reduction percentages were 67.71%, 79.11%, and 85.84%, respectively). A total of 42.5% of patients achieved EASI75 at week 4, 69.7% at week 16, while at week 52, the percentage of patients who achieved EASI75 raised to 82.35%.

The mean DLQI score was 18.15 at baseline and it decreased to 7.8 at week 4, to 5.85 at week 16, and to 3.59 at week 52 (reduction percentages were 57.02%, 67.77%, and 80.22%, respectively).

The mean itch-NRS score was 8.61 at baseline and it decreased to 4 at week 4, to 3.12 at week 16, and it increased to 3.23 at week 52 (reduction percentages were 53.54%, 63.76%, and 62.48%, respectively).

The mean sleep-NRS score was 7.61 at baseline and it decreased to 2.2 at week 4, to 1.27 at week 16, and to 1.06 at week 52 (reduction percentages were 71.09%, 83.31%, and 86.07%, respectively).

#### 3.2.3. Generalized Eczema with a Lichenoid Pattern (42 Patients)

The mean EASI score was 31.14 at baseline and it decreased to 8.53 at week 4, to 4.63 at week 16, and it slightly increased to 6.82 at week 52 (reduction percentages were 72.61%, 85.13%, and 78.1%, respectively). A total of 63.16% of the patients achieved EASI75 at week 4, 81.48% at week 16, while at week 52, the percentage of patients who achieved EASI75 raised to 90.91%.

The mean DLQI score was 15.74 at baseline and it decreased to 6.39 at week 4, to 4.26 at week 16, and to 1.6 at week 52 (reduction percentages were 59.4%, 72.93%, and 89.83%, respectively).

The mean itch-NRS score was 8.79 at baseline and it decreased to 4.05 at week 4, to 3.07 at week 16, and to 2.3 at week 52 (reduction percentages were 53.92%, 65.07%, and 73.83%, respectively).

The mean sleep-NRS score was 6.9 at baseline and it decreased to 1.74 at week 4, to 1.41 at week 16, and to 0 at week 52 (reduction percentages were 74.78%, 79.56%, and 100%, respectively).

#### 3.2.4. Prurigo (18 Patients)

The mean EASI score was 27.94 at baseline and it decreased to 13.53 at week 4, to 6.14 at week 16, and to 3.67 at week 52 (reduction percentages were 51.57%, 78.02%, and 86.86%, respectively). Only 20% of patients achieved EASI75 at week 4, while at week 16, the percentage of patients who achieved EASI75 raised to 64.29%, and at week 52, EASI75 was reached by 83.33% of patients.

The mean DLQI score was 13.83 at baseline and it decreased to 5.4 at week 4, to 4.5 at week 16, and to 2.92 at week 52 (reduction percentages were 60.95%, 67.46%, and 78.89%, respectively).

The mean itch-NRS score was 8.28 at baseline and it decreased to 4.47 at week 4, to 2.93 at week 16, and to 2.83 at week 52 (reduction percentages were 53.92%, 65.07%, and 73.83%, respectively).

The mean sleep-NRS score was 6.44 at baseline and it decreased to 2.67 at week 4, to 0.5 at week 16, and to 0 at week 52 (reduction percentage were 58.54%, 92.24%, and 100%, respectively).

#### 3.2.5. Nummular Eczema (Eight Patients)

The mean EASI score was 31.87 at baseline and it decreased to 11 at week 4, to 6.83 at week 16, and it increased to 7 at week 52 (reduction percentages were 65.48%, 78.57%, and 78.04%, respectively). Only 16.67% of patients achieved EASI75 at week 4, the percentage of patients who achieved EASI75 raised to 83.33% at week 16, while at week 52, the percentage of patients who achieved EASI75 slightly decreased to 75%.

The mean DLQI score was 16.37 at baseline and it decreased to 4.5 at week 4, it slightly increased to 6.33 at week 16, and it improved to 3.75 at week 52 (reduction percentages were 72.51%, 61.33%, and 77.09%, respectively).

The mean itch-NRS score was 8.37 at baseline and it decreased to 4.17 at week 4, to 3.67 at week 16, and it increased to 5.25 at week 52 (reduction percentages were 50.18%, 56.15%, and 37.28%, respectively).

The mean sleep-NRS score was 6.12 at baseline and it decreased to 3.67 at week 4, to 1.5 at week 16, and to 1.25 at week 52 (reduction percentages were 40.03%, 75.49%, and 79.57%, respectively).

#### 3.2.6. Erythroderma (11 Patients)

The mean EASI score was 39 at baseline and it decreased to 14.44 at week 4, to 7.22 at week 16, and to 5.87 at week 52 (reduction percentages were 62.97%, 81.49%, and 84.95%, respectively). A total of 55.56% of patients achieved EASI75 at week 4, 77.78% at week 16, while at week 52, the percentage of patients who achieved EASI75 slightly decreased to 75%.

The mean DLQI score was 16.91 at baseline and it decreased to 8.56 at week 4, to 2.89 at week 16, and to 2 at week 52 (reduction percentages were 49.38%, 82.91%, and 88.17%, respectively).

The mean itch-NRS score was 8.82 at baseline and it decreased to 4.11 at week 4, to 1.78 at week 16, and it slightly increased to 2.25 at week 52 (reduction percentages were 53.4%, 79.82%, and 74.49%, respectively).

The mean sleep-NRS score was 6.82 at baseline and it decreased to 2.67 at week 4, to 0.67 at week 16, and to 0 at week 52 (reduction percentages were 60.85%, 90.18%, and 100%, respectively).

### 3.3. Statistical Analysis for the Different Clinical Phenotypes

We performed ANOVA tests to evaluate the EASI score at the beginning of the therapy in the six AD phenotypes and we found that the mean EASI score in the generalized patterns and erythrodermic pattern was significantly higher than the mean EASI score in the classic phenotype (inflammatory (*p* = 0.003), lichenoid (*p* = 0.012), and erythrodermic (*p* < 0.0001)). We did not find the same differences in the other evaluated scores (DLQI, itch NRS, sleep NRS). Moreover, we performed a paired *t*-test to evaluate the reduction of all the scores after 16 weeks of treatment and their statistical significance, and we found a *p*-value < 0.0001 for all the scores. We did not perform the statistical analysis after 52 weeks from the beginning of the therapy because of the low number of patients (72 patients, 32.58%) who reached 52 weeks of treatment.

## 4. Discussion

The incidence of AD is increasing steadily, especially in industrialized countries. The diagnostic criteria for this disease are basically clinical and well standardized in children [6]. The diagnosis can be a challenge when a dermatosis clinically resembling AD manifests in an adult without a history of AD or atopy. Moreover, in adults, the clinical and morphological characteristics of AD differ from those seen in children. Thus, it is a diagnosis of exclusion that we can only reach after performing patch testing, blood analysis, and/or a cutaneous biopsy to rule out other diseases. In a recent paper, Silvestre et al. [19] reported the different clinical phenotypes of adult AD by describing their specific characteristics. In our study, we evaluated 221 adult patients affected by severe AD and treated with dupilumab; we found that 60 (27.15%) of them had adult-onset AD. In a previous paper of Hello et al. [14] in 2016, the percentage of adult-onset AD was reported as 18%. The higher percentage that we found was probably related to the fact that we studied a cohort of selected patients with moderate or severe AD candidates for therapy with dupilumab. In our cohort of patients, the most frequent clinical phenotype was the classic one, manifesting as lichenified/exudative flexural dermatitis, which was almost always associated with head-and-neck eczema and/or hand eczema, as previously described [14]. In the present study, the classic AD phenotype was present with the same distribution in the total, early-onset, and adult-onset subgroup patients (45.7%, 45.95%, and 45%, respectively).

Interestingly, the prurigo phenotype was much more frequent in adult-onset than in early-onset AD (13 patients (21.67%) versus 5 patients (3.11%), respectively), while the erythroderma phenotype was more common in early-onset than in adult-onset AD (1 patient (1.66%) versus 10 patients (6.21%), respectively).

Overall, 75.97% of our patients achieved EASI75 at week 16 of the dupilumab treatment and 83.33% achieved EASI75 at week 52, which are percentages higher than those reported in the registration studies (65% in LIBERTY AD CHRONOS and 67% in LIBERTY AD CAFE, respectively) [17,18].

We evaluated the EASI score of the six AD phenotypes at baseline and found that the generalized inflammatory and lichenoid phenotypes and the erythrodermic phenotype all showed an EASI that was significantly higher than that of the classic phenotype. In contrast, the other scores, including DLQI, itch-NRS, and sleep-NRS, did not show any significant difference between phenotypes at baseline, suggesting that AD had a high impact on the patients’ quality of life regardless of the different clinical presentations.

At week 4, a good clinical response was seen in all the phenotypes but with differences between them since the highest reduction (76%) of the EASI score was achieved in the classic phenotype and the lowest one in the prurigo phenotype (52%). At week 16, an 80% reduction in the EASI score was reached in all the phenotypes and this response was maintained after 52 weeks of treatment. It is worthy of note that the prurigo and nummular eczema phenotypes responded more slowly than the other phenotypes, but after 16 and 52 weeks of treatment, these two phenotypes reached an EASI75 similar to those of the other ones.

Concerning the improvement in the patients’ quality of life at the different time points of the dupilumab treatment, all the clinical phenotypes showed a statistically significant DLQI reduction compared to the baseline. Interestingly, the highest DLQI reduction was obtained at week 52 in the generalized lichenoid and erythroderma phenotypes, which were the settings showing the higher DLQI scores at baseline.

When evaluating the impact of the AD on the quality of life of the patients, we also considered the incidence of pruritus and the quality of the sleep. After 4 weeks of therapy, at least a 50% reduction in NRS was achieved for all the phenotypes, with further improvement at week 16 that persisted to week 52. Consequently, the best improvement was seen in the erythroderma setting, in line with the results observed for DLQI.

Another important parameter assessed was the sleep NRS score. We found a good improvement (70% of the baseline score) in all six phenotypes already at week 4, reaching a 100% reduction after 52 weeks of treatment in three of the six phenotypes (generalized lichenoid, prurigo, and erythroderma).

## 5. Conclusions

Our study supports the efficacy and safety of dupilumab in the treatment of severe atopic dermatitis given the findings of rapidly improving signs and symptoms, including skin manifestations, such as erythema, edema/papulation, excoriation and lichenification, and pruritus, as well as sleep disturbances and the overall patients’ quality of life. Our results at week 52 show that dupilumab used in monotherapy or in combination with topical corticosteroids was a choice agent for long-term management of AD. In all six AD phenotypes, a good response in terms of clinical improvement and quality of life impact was observed. Of note, in the prurigo and nummular eczema phenotypes, the EASI reduction was slower than in the other phenotypes, which suggests that treatment should not be discontinued until reaching a satisfactory clinical response.

This study has two limitations: the first one is related to its retrospective nature and the second one is the low number of patients reaching week 52 of the dupilumab treatment. The strength of the study is in the stratification of AD in six different phenotypes based on patients’ clinical presentation, where all of which responded well to dupilumab in the assessed scores; it was noteworthy that the most severe phenotypes at the baseline, notably erythroderma, recorded the best improvement in terms of both the clinically evident and reported symptoms.

## Figures and Tables

**Table 1:** The mean Eczema Area and Severity Index (EASI) score at baseline for classic AD phenotype when compared to the other groups.

Group	Group	*p*-Value	95% Confidence Interval (CI)	
			Lower Limit	Upper Limit
1	2	0.003	−6.4972	−1.3437
	3	0.012	−5.8377	−0.728
	4	0.963	−3.6421	3.4732
	5	0.123	−9.1203	1.0903
	6	0.000	−15.5539	−6.7261

Legend: 1 Classic form, 2 Generalized inflammatory form, 3 Generalized lichenoid form, 4 Prurigo, 5 Nummular eczema form, 6 Erythroderma.

**Table 2:** Number of patients subgrouped into the six clinical phenotypes of AD.

Clinical Phenotype	Total Number of Patients (% of 221 Patients)	Number of Patients with Early-Onset AD (% of 161 Patients with Early-Onset AD)	Number of Patients with Adult-Onset AD (% of 60 Patients with Adult-Onset AD)
Classic: Lichenified/exudative flexural dermatitis, almost always associated with head-and-neck eczema and/or hand eczema	101 (45.7%)	74 (45.95%)	27 (45%)
Generalized eczema with an inflammatory pattern	41 (18.55%)	34 (21.12%)	7 (11.67%)
Generalized eczema with a lichenoid pattern	42 (19%)	33 (20.5%)	9 (15%)
Prurigo	18 (8.14%)	5 (3.11%)	13 (21.67%)
Nummular eczema	8 (3.63%)	5 (3.11%)	3 (5%)
Erythroderma	11 (4.98%)	10 (6.21%)	1 (1.66%)
Total number of patients	221	161	60

**Table 3:** EASI score, EASI75, and Dermatology Life Quality Index (DLQI) of the 221 treated with dupilumab at baseline and after 4, 16, and 52 weeks from the beginning of the treatment.

Timepoints	Number of Patients (%)	EASI (% Reduction)	EASI75	DLQI (% Reduction)
Baseline	221	30.73		16.41
4 weeks	221 (100%)	8.66 (71.03%)	49.77% (110 patients)	7.02 (57.74%)
16 weeks	154 (69.68%)	5.29 (82.4%)	75.97% (117 patients)	4.72 (71.46%)
52 weeks	72 (32.58%)	4.59 (84.64%)	83.33% (60 patients)	2.8 (83.14%)
